# Interactions between Damaged Hair Keratin and Juglone as a Possible Restoring Agent: A Vibrational and Scanning Electron Microscopy Study

**DOI:** 10.3390/molecules29020320

**Published:** 2024-01-09

**Authors:** Michele Di Foggia, Paola Taddei, Carla Boga, Benedetta Nocentini, Gabriele Micheletti

**Affiliations:** 1 Department of Biomedical and Neuromotor Sciences, Alma Mater Studiorum-Università di Bologna, Via Irnerio 48, 40126 Bologna, Italy; michele.difoggia2@unibo.it; 2 Department of Industrial Chemistry ‘Toso Montanari’, Alma Mater Studiorum-Università di Bologna, Via Piero Gobetti 85, 40129 Bologna, Italy; carla.boga@unibo.it (C.B.); gabriele.micheletti3@unibo.it (G.M.); 3 Kemon S.p.A., Via Enrico Mattei 35, 06016 San Giustino, Italy; benedetta.nocentini@gmail.com

**Keywords:** hair keratin, juglone, repairing agent, thia-Michael, bleaching, reduction, methyl thioglycolate, sodium hydrogen sulfite, IR spectroscopy, Scanning Electron Microscopy

## Abstract

Juglone, a quinonic compound present in walnut extracts, was proposed as a restoring agent for hair keratin treated with permanent or discoloration processes. The proposed mechanism of restoration by juglone involves the formation of a Michael adduct between the quinone and the thiol moieties of cysteine residues. To this purpose, the first part of the present paper involved the spectroscopic study of the product of the reaction between juglone and N-acetyl-L-cysteine as a model compound. IR spectroscopy and Scanning Electron Microscopy (SEM) monitored the chemical and morphological variations induced by applying juglone to hair keratin. In order to simulate the most common hair treatments (i.e., permanent and discoloration), juglone was applied to hair that had been previously treated with a reducing agent, i.e., methyl thioglycolate (MT) or with bleaching agents (based on hydrogen peroxide and persulfates) followed by sodium hydrogen sulfite. IR spectroscopy allowed us to monitor the formation of Michael adducts between juglone and cysteine residues: the Michael adducts’ content was related to the cysteine content of the samples. In fact, MT and sodium hydrogen sulfite favored the reduction of the disulfide bonds and increased the content of free cysteine residues, which can react with juglone. SEM analyses confirmed the trend observed by IR spectroscopy since hair samples treated with juglone adopted a more regular hair surface and more imbricated scales, thus supporting the possible use of juglone as a restoring agent for damaged hair keratins.

## 1. Introduction

The cuticle is the external structure of hair [1] and consists of flattened cells that protect the inner layers (cortex and medulla) from external agents and adopts a prevalent β-sheet conformation [2]. From a chemical point of view, the hair cuticle is mainly made up of keratin (65–95%), lipids (mostly in the form of 18-methyleicosanoic acid or 18-MEA [3], 1–9%), and pigments (0.3–0.9%). The cuticle is the layer with the highest cystine concentration and thus of disulfide bridges [2]; free cysteines provide the thioester bond with a protective layer rich in 18-MEA [4].

Cosmetic alterations of hair allow for the modification of its shape or color. From a structural point of view, permanent treatments involve the application of an alkaline solution capable of opening the cells of the cuticle to favor the penetration of a reducing agent capable of breaking the disulfide bonds [2,5]. The reduction treatment can be performed with sulfites, bisulfites (softer), or mercaptans (stronger, for example, thioglycolates). In the treatment with bisulfites (sulfitolysis), disulfide bonds are cleaved to give a cysteine thiolate (K-S^−^, where K indicates keratin) and a cysteine-S-sulfonate or Bunte salt (K-S-SO_3_^−^). In the case of thioglycolate, the alkaline environment is needed to induce several effects: an increase in the concentration of the active thiolate ion (RS^−^), which can contribute to causing unwanted reactions in the hair, such as the hydrolysis of peptide bonds [6], and an increase in the reduction potential, so that the reduction rate of thioglycolate can be higher than the diffusion rate into hair [7]. The subsequent oxidative treatment to oxidize unreacted cysteine and mercaptan residues contributes to protein fragmentation, resulting in the loss of cystines and an increase in cysteic acid. The oxidative pathway of cystines to cysteic acid takes place through the formation of the intermediate species cystine-S-monoxide and cystine-S-dioxide, which are more reactive than the parent disulfide [8].

The hair discoloration treatment also involves oxidation in an alkaline environment: these conditions allow the destruction of the chromophore groups of the melanin pigments inside the cortex. Under oxidizing conditions, the agglomerates of melanin, linked to keratin through polypeptide residues, depolymerize, leaving carboxylic derivatives very soluble in water. The breaking of the disulfide bonds favors access to the melanin granules and leads to the formation of oxidized derivatives that are more sensitive to splitting in an alkaline environment.

From what has been said, cosmetic hair treatments and environmental stress can increase the concentration of cysteine residues containing the free thiol group. The latter can give Michael addition by acting as donors (as nucleophiles) towards acceptors (electrophiles) such as α,β-unsaturated carbonyls. For example, juglone (5-hydroxy-1,4-naphthoquinone) is a potent electrophile capable of reacting with glutathione in keratinocytes, causing its depletion and explaining its cytotoxicity [9]. The same reaction can, however, be exploited to restore the damaged hair’s external structure because it involves the free thiol groups. Michael acceptors, such as shikimic acid and several maleic acid derivatives, are present in the formulations of commercial products with a restructuring action [10]. Juglone is the primary coloring component of walnut (*Juglans regia* L.) extracts and has been used as a brown dye of natural (wool, cotton, and silk) or artificial (nylon) fabrics [11]. Moreover, it has been proposed as a natural semi-permanent hair dyeing agent with interesting anti-microbial properties that induces low skin irritation [12,13].

To gain information about the possibility of using juglone as a restoring agent, hair samples were subjected to different chemical stresses, simulating the most common hair treatments, i.e., permanent and bleaching. For the former process, a juglone methanolic solution was applied to hair samples previously treated with an alkaline solution and a reducing agent to simulate the first step of the permanent process. The chosen reducing agent was methyl thioglycolate, a thioglycolic acid ester, which is less used in cosmetic treatment than ammonium thioglycolate but has lower risks of skin irritation [14]. For the latter process, juglone was applied to hair subjected to alkaline and repeated bleaching processes (i.e., three bleaching treatments, which are the most common in hairdresser saloons) as previously conducted for the study of other formulations based on unsaturated Michael acceptors such as shikimic acid and bis-aminopropyl diglycol dimaleate [10]. Moreover, juglone was also applied to hair samples further treated with sodium hydrogen sulfite to mimic a straightening treatment following bleaching. The samples were characterized at each step of the study using vibrational Attenuated Total Reflectance (ATR)/Fourier Transform (FT)-IR Spectroscopy and Scanning Electron Microscopy (SEM). The ATR technique has proved extremely useful in characterizing the surface chemistry of many substrates, such as wool or hair fibers (particularly the hair cuticle layer) [15], with the additional advantages of being non-destructive and not requiring any sample manipulation. The present study is aimed at spectroscopically investigating the reactivity of the cysteine residues with juglone in deteriorated hair by exploring the possible formation of Michael addition products. For this purpose, the products of the model reaction between juglone and N-acetyl cysteine, under mild reaction conditions [16], were characterized using ATR/IR spectroscopy. The use of polyphenols (such as juglone) as hair-repairing agents has been previously explored since they can bind to proteins (i.e., by the above-mentioned Michael reactions) and may be used as anchoring spacers with conventional hydrophobic molecules (i.e., long alkyl quaternary ammonium salts) which favor the restoration of the lipidic coating of hair [4].

## 2. Results and Discussion

### 2.1. IR Analyses

#### 2.1.1. Model Compounds

**Figure 1:**
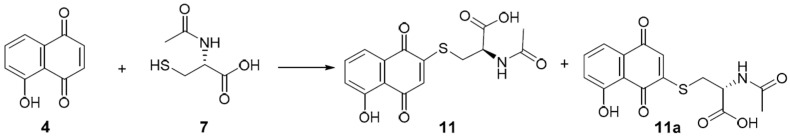
Scheme of the reaction between juglone and N-acetyl-L-cysteine [16].

A previous paper by Micheletti et al. [16] studied the reaction (Michael addition) between N-acetyl-L-cysteine and juglone, resulting in the products shown in Figure 1. 

**Figure 2:**
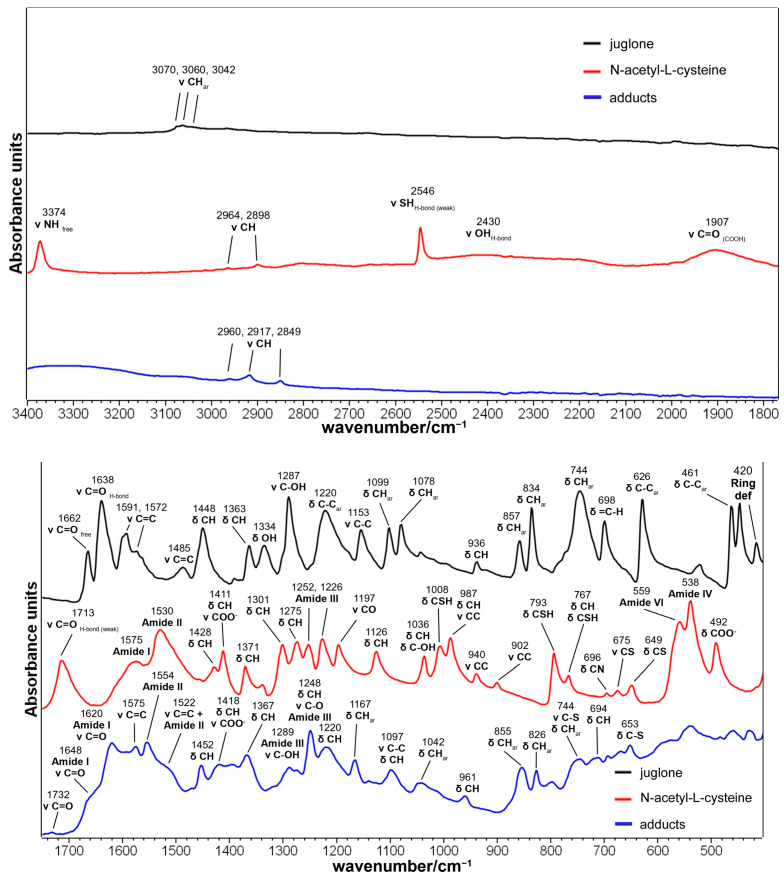
IR spectra of juglone (black line), N-acetyl-L-cysteine (red line), and their adducts (blue line) in the 3400–1750 and 1750–400 cm^−1^ spectral ranges. Table S1 (Supplementary Materials) reports the references for the assignments of the main IR bands.

N-acetyl-L-cysteine was chosen as a model compound for proteins; the acetyl group also prevents the aza-Michael reaction with the amino group [17], thus maximizing the yield of the desired thia-Michael reaction. The IR spectra of juglone (5-hydroxynaphtalene-1,4-dione), N-acetyl-L-cysteine, and their adducts are shown in Figure 2: bands attribution (Table S1, Supplementary Materials) was performed based on the previous literature (juglone [18,19,20,21,22,23,24,25,26]; N-acetyl-L-cysteine [27,28,29,30,31]; adducts [25,32,33,34]). The disappearance of the S-H stretching band at 2546 cm^−1^ in the adduct spectrum (Figure 2) indicates the complete reaction of N-acetyl-L-cysteine: the intensity of this band was used by Long et al. [33] to monitor the kinetics of thia-Michael reactions between mono- and difunctional thiols and alkenes. Moreover, other bands attributed to the thiol group (i.e., 1008, 793, and 767 cm^−1^ [31]) were absent in the adduct spectrum (Figure 2). The adduct formation can be further supported by the 961 and 855 cm^−1^ bands, assigned to the out-of-plane deformation of the CH and CH_2_ groups of unsaturated organic sulfur compounds [35]. The former band was absent either in juglone or in N-acetyl-L-cysteine spectra, while the latter has a contribution from the aromatic bending vibration of CH in juglone at 857 cm^−1^ (Figure 2); nevertheless, in the adduct spectrum, the band is wider (FWHM increases from 11 to 19 cm^−1^), thus suggesting the presence of an additional spectral component.

The IR spectra offered an insight into the redox state of the products through the presence of the 1648 and 1620 cm^−1^ bands attributed to the stretching vibration of the carbonyl groups of quinones. This observation confirmed that the thia-Michael reaction between quinones and mercaptans should lead to the formation of hydroquinonic products [16], which may quickly oxidize to quinones via air oxidation or because of non-reacted quinones, which leads to a reduced yield of the addition [16]. 

#### 2.1.2. Brown Hair Treated with KOH and Methyl Thioglycolate

**Figure 3:**
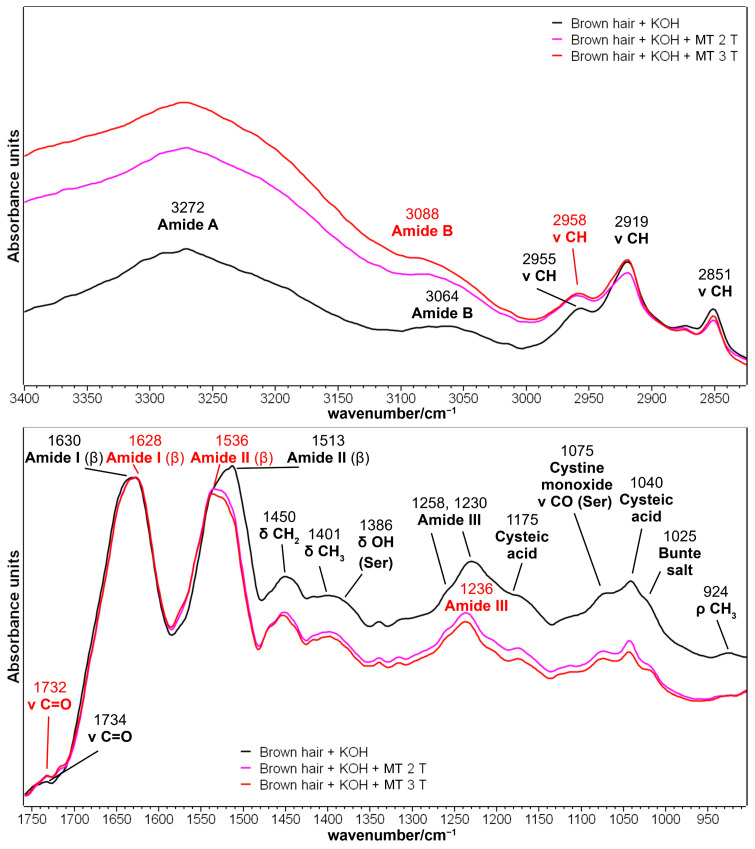
IR spectra of brown hair lock after the treatment with aqueous KOH (pH 9) for 5 min (black line) and KOH + methyl thioglycolate (MT) for two and three times (2 T and 3 T, magenta and red lines, respectively) in the 3400–2800 and 1760–900 cm^−1^ spectral ranges. Spectra are normalized to the Amide I band. Abbreviations: β: β-sheet structure; Ser: Serine.

Figure 3 reports the IR spectra in the 3400–2800 and 1760–900 cm^−1^ ranges of brown hair after treatment with aqueous KOH alone (pH 9, 5 min), and KOH plus the methyl thioglycolate (MT)-reducing agent up to three times. The treatment with KOH or other strong basic agents in hair treatment is widespread due to the lability of disulfide bonds in alkaline conditions, with the occurrence of the so-called lanthionization reaction, which causes the conversion of disulfide bonds (-CH_2_-S-S-CH_2_-) into stable lanthionine bonds (-CH_2_-S-CH_2_-) [36]. However, the treatment used in the present study did not affect the cuticle structure as previously reported under similar conditions (treatment with KOH for 5 min at pH 8.7 [37,38]); therefore, the alkali-treated hair was considered the control sample. Other authors [39] observed that alkaline treatments were less effective in inducing structural changes in keratin compared with acidic ones. The spectral comparison between the control sample (brown hair + KOH) and virgin hair is reported in Figure S1 (Supplementary Materials): the weakening of CH stretching vibrations at about 2920–2850 cm^−1^ can be related to the depletion of the epicuticle (the outermost lipid layer of the cuticle that includes 18-MEA and free lipids [5]) due to the use of a strong base [40] and further supported by the weakening of the carbonyl stretching band attributed to carboxylic acids and esters at 1736 cm^−1^. Another slight decrease was observed around 1032 cm^−1^ and attributed to both cysteic acid and Bunte salt depletion; a previous paper reported the complete removal of Bunte salt in more severe alkaline conditions (i.e., pH = 9.5, 48 h of application) [41]. 

The control sample (brown hair + KOH) has an overall β-sheet structure as indicated by the position of the Amide I, II, and III peaks (1630, 1513, and 1230 cm^−1^, respectively) and corresponding to the cuticle layer, i.e., the outer layer of a hair’s keratin analyzable with the ATR technique [37,38]. Besides Amide bands sensitive to the secondary structure of the cuticle, this layer is rich in serine residues, which showed characteristic IR bands at 1386 cm^−1^ (δ OH, mixed with a CH_3_ bending vibration) and 1075 cm^−1^ (ν CO) [37,38].

Thioglycolates (in particular ammonium thioglycolate) are reducing agents commonly used in hair straighteners to reduce disulfide bonds to thiols, allowing the mechanical relaxation of keratin [42]. In the present study, the used straightener was MT: its application on control brown hair mainly affected the 1250–950 cm^−1^ spectral region, where the vibrations attributed to sulfur oxidation products have been reported to fall [8]: cysteic acid, cystine monoxide, cystine dioxide, and Bunte salt (Figure 3 and Figure S2 and Table 1); therefore, the trend of the spectra testifies the effectiveness of the reducing treatment. The chemical structure of the above-mentioned sulfur compounds is reported in Table S2 (Supplementary Materials) as a reader’s help. The relative content of cysteic acid can be estimated from the intensity ratios between the bands attributed to cysteic acid (i.e., 1175 and 1040 cm^−1^) and the Amide I band (taken as an internal standard); the values of the I1175/I_Amide I_ and I1040/I_Amide I_ ratios are reported in Table 1. Both intensity ratios showed a significant reduction after two treatments with the reducing agent; the further application of MT (third time) also reduced cysteic acid content significantly (Table 1). 

Bunte salts (alkyl or aryl thiosulfates, Table S2, Supplementary Materials) are another class of sulfur oxidation products whose relative content can be estimated by the area ratio between the 1025 cm^−1^ band (attributed to those compounds) and the 1040 cm^−1^ band previously attributed to cysteic acid. The values of the A1025/A1040 ratio reported in Table 1 show that the content of the Bunte salts decreased with respect to cysteic acid (although not significantly) after the second application of MT and remained stable in the case of further application (Table 1). This observation was supported by the 1199 and 1034 cm^−1^ negative peaks in Figure S2 (Supplementary Materials) in the difference spectrum and attributed to Bunte salts vibrations [8]. For this purpose, it may be recalled that Erra et al. [41] described the complete removal of Bunte salts after treating wool fibers with 25 g/L of ammonium thioglycolate for 1 h at room temperature. Conversely, increased cysteic acid content, compared with Bunte salts, was reported after the prolonged light exposure of wool [43].

Unfortunately, it was impossible to observe any increase in the S-H stretching band at about 2550 cm^−1^ following MT treatments. However, the reduction of disulfide bridges can be inferred by the spectral differences in the 1600–1475 cm^−1^ spectral region (Figure 3 and Figure S2). In particular, brown hair treated with MT showed an increased intensity in the 1590–1550 cm^−1^ region: the difference spectrum in Figure S2 (Supplementary Materials) showed a positive peak at 1562 cm^−1^, corresponding to a band diagnostic of cysteine [44]. The decreased intensity in the 1530–1475 cm^−1^ of the treated hair (corresponding to a negative peak at 1497 cm^−1^ in the difference spectrum, Figure S2, Supplementary Materials) may be attributed to the 1490 cm^−1^ band, diagnostic of cystine and previously used by Cataldo et al. [44] to monitor the oxidation of cysteine into cystine induced by gamma-radiation. In addition, the negative peak at 1658 cm^−1^ can be attributed to the carbonyl stretching vibration of cystine [45], thus confirming the efficacy of MT in breaking hair disulfide bridges. Applying a solution of 6% thioglycolic acid at pH 9 at room temperature for 5 min penetrated the cuticle layer of hair entirely and induced the disconnection of 90% of disulfide bridges [2]. Fernandez-d’Arlas et al. [36] suggested that using thioglycolates, beyond its reductive power, also favors incorporating ionisable carboxylic groups into keratin: the positive band at 1562 cm^−1^ observed in the difference spectrum in Figure S2 (Supplementary Materials) could also be attributed to the asymmetric stretching vibration of carboxylate groups. 

In general, the reducing agents poorly affected the secondary structure of treated hair, as previously observed on wool fibers treated with ammonium and calcium thioglycolate [42,46]. The differences observed in the I_Amide I_/I_Amide II_ absorbance ratio (Table 1) after the thioglycolate application can be better related to the cystine reduction to cysteine, discussed in the previous paragraph, rather than a structural rearrangement. This hypothesis can be further supported by the FWHM of both Amide I and II bands, which remained almost unchanged (67 and 56 cm^−1^, respectively). 

#### 2.1.3. Brown Hair Treated with KOH, Methyl Thioglycolate, and Juglone

**Figure 4:**
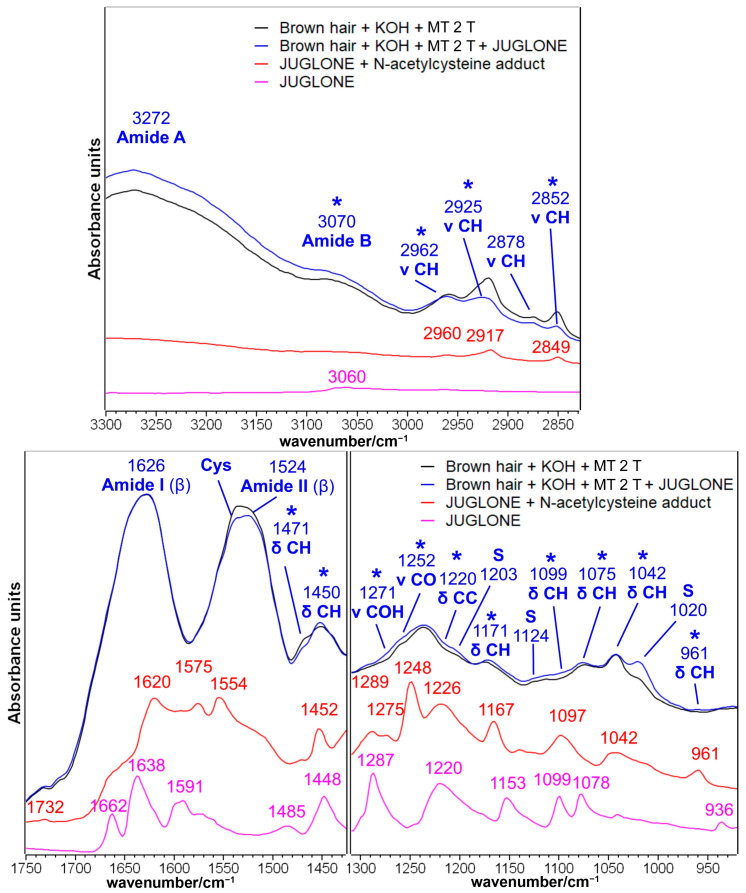
IR spectra of brown hair lock treated with KOH and methyl thioglycolate (MT) for two times (black line) and after the following treatment with a methanolic juglone solution (blue line) in the 3300–2800 and 1750–920 cm^−1^ spectral ranges. Spectra are normalized to the Amide I band. The IR spectra of pure juglone (magenta line) and the thia-Michael addition product between juglone and N-acetyl-L-cysteine (red line) are reported for comparison (see Figure 2 for more details). Bands marked with an asterisk are related to juglone incorporation; S: bands attributed to sulfur oxidation products.

**Figure 5:**
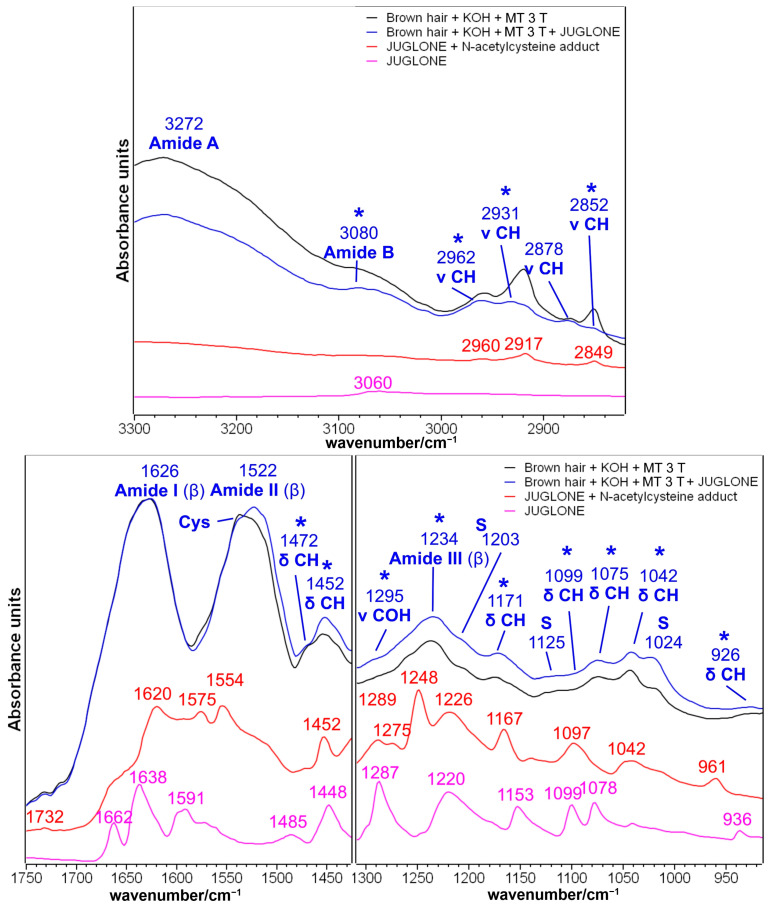
IR spectra of brown hair lock treated with KOH and methyl thioglycolate (MT) for three times (black line) and after the following treatment with a methanolic juglone solution (blue line) in the 3300–2800 and 1750–910 cm^−1^ spectral ranges. Spectra are normalized to the Amide I band. The IR spectra of pure juglone (magenta line) and the thia-Michael addition product between juglone and N-acetyl-L-cysteine (red line) are reported for comparison (see Figure 2 for more details). Bands marked with an asterisk are related to juglone incorporation; S: bands attributed to sulfur oxidation products.

Juglone (5-hydroxynaphtalene-1,4-dione) is a naphthoquinone that can be extracted from black walnut (*Juglans regia* L.) and used as a reddish-brown natural dye [11]. The IR spectra before and after the treatment with the natural dye after the second and third reduction with MT are presented in Figure 4 and Figure 5, respectively. The spectral differences in the 1750–900 cm^−1^ spectral region may suggest that the juglone chemically interacted with the hair cuticle. The treated hair (Figure 4 and Figure 5) showed an increased absorbance in several spectral regions, which corresponded well with the main bands observed in the adduct between the juglone and the cysteine, which was previously considered as a model compound for the chemical interaction by a thia-Michael reaction between the juglone and cysteine residues of hair keratin [16]: 1452–1450, 1295–1271, 1252, 1220, 1171, 1099, 1075, 1042 and 961–926 cm^−1^ (see attributions in Figure 2 and Table S1, Supplementary Materials). A similar mechanism of reaction (i.e., the nucleophilic addition of SH groups to juglone) was proposed by Inbaraj et al. who explained the cytotoxicity of juglone towards keratinocytes, which depleted cells of glutathione [9].

Besides the chemical interaction between hair keratin and juglone through the Michael reaction, naphthoquinone may act as an electron transfer agent in oxidation-reduction reactions [47]; in particular, juglone is a pro-oxidant molecule since it can transfer electrons from a biological substrate to oxygen, generating a moderate quantity of reactive oxygen species (ROS) that can oxidize functional groups on proteins [47,48]. More in detail, thiol groups of cysteine residues can be oxidized by this mechanism to sulfenic acid (R-SOH), sulfinic acid (R-S(O)OH), and sulfonic acid (R-S(O)_2_OH [47]. This redox mechanism could explain the most evident variation observed in the IR spectra of juglone-treated hair, i.e., the strengthening of the IR band at 1024–1020 cm^−1^ (Figure 4 and Figure 5); this spectral feature may be assigned to Bunte salt as well as to Cys-sulfinate salts [49]. The oxidation of sulfur atoms induced by juglone may also explain the increased absorptions at 1203 cm^−1^ (Bunte salt) and 1125–1124 cm^−1^ (cystine dioxide), while the formation of the other sulfur oxidation species, such as cysteic acid (1175 and 1040 cm^−1^) and cystine monoxide (1075 cm^−1^), could only be hypothesized because of the overlapping with some of the above-mentioned bands of the thia-Michael products (Figure 4 and Figure 5). This overlapping suggested not calculating the I_1175_/I_Amide I_, I_1040_/I_Amide I_, and A1025/A1040 ratios to estimate the cysteic acid and Bunte salt contents. The same oxidation process may explain the decreased absorbance of the spectral region between 1560 and 1530 cm^−1^, where a diagnostic band of cysteine is present [44] (Figure 4 and Figure 5). It must be recalled that the pristine application of MT, a reducing agent, induced an opposite effect on the content of both cysteine residues and Bunte salts, as shown in Figure 3 and Figure S2.

Interestingly, in the CH stretching region (i.e., 3000–2800 cm^−1^, Figure 4 and Figure 5), the IR spectrum of juglone-treated hair showed a decreased absorbance. Pure juglone does not show any peak in this region (Figure 2); therefore, this trend may be interpreted by considering that, as a result of juglone incorporation onto the surface of the hair fiber, the spectral contribution of the lipidic component decreased. Accordingly, the only other band decreasing after the treatment with juglone is the 1472–1470 cm^−1^ spectral feature, which is attributed to the bending vibration of the CH groups in 18-MEA [50].

Besides cysteine, i.e., the most reactive amino acid, it cannot be excluded that other amino acids participate in Michael addition reactions, particularly lysine, histidine, and serine (in decreasing order of reactivity) [47,51]. Serine is the second most common amino acid in human hair (672–1130 μM/g), while lysine and histidine showed lower contents (178–236 μM/g and 56–85 μM/g, respectively) [52]. The reaction between juglone and Lys or His residues’ side chains will convert the primary amine into a secondary amine group following the mechanism proposed in Figure S3, Supplementary Materials [51], and show bands close to 1171 and 1099 cm^−1^ (Figure 4 and Figure 5), which may also have a contribution from the C-N stretching vibration and N-CH bending, respectively [35].

The effects of juglone on hair samples treated three times with MT were similar (Figure 5) to those described in the previous paragraph, but the spectral differences were more pronounced when compared with hair subjected to only two reducing steps (Figure 4). In particular, the formation of the Michael adduct between keratin and juglone could be inferred by the spectral variations of the bands at 2962, 2931, 2852, 1452, 1171, 1099, 1075, 1042, and 926 cm^−1^. Other juglone-related bands were observed in the spectral regions of Amide B (i.e., 3080 cm^−1^) and Amide III (i.e., 1295 and 1234 cm^−1^). Moreover, the intensity increase observed for the bands attributed to sulfur oxidation products (i.e., 1203, 1125, and 1024 cm^−1^) was more evident compared with what was observed in Figure 4 for samples treated twice with MT, mainly because the third reducing treatment proved to be effective towards the content of cysteic acid (Table 1). The Amide II region between 1600 and 1500 cm^−1^ appeared to be the most affected by the treatment with juglone. Also in this case, the observed variations can be attributed to the diagnostic bands of cysteine (i.e., 1530 cm^−1^) and cystine (i.e., 1495 cm^−1^) as a consequence of the pro-oxidant effect of juglone, rather than to structural variations in the secondary structure of hair keratin, since the Amide I band appeared almost unaffected by the treatment.

#### 2.1.4. Brown Hair Bleached Three Times and Treated with Juglone

The effects of three bleaching treatments on hair keratin with the hair products used in the present study were discussed in detail in a previous publication [53]. Briefly, as sulfur oxidation products (i.e., mainly cysteic acid and Bunte salts, but also cystine monoxide and dioxide) increased, the secondary structure of hair keratin became more disordered as revealed by the shift in the structural-sensitive bands (i.e., Amide II, Amide A, and Amide B) and serine bands decreased due to the involvement of this amino acid in the degradation processes induced by bleaching with H_2_O_2_ [53].

**Figure 6:**
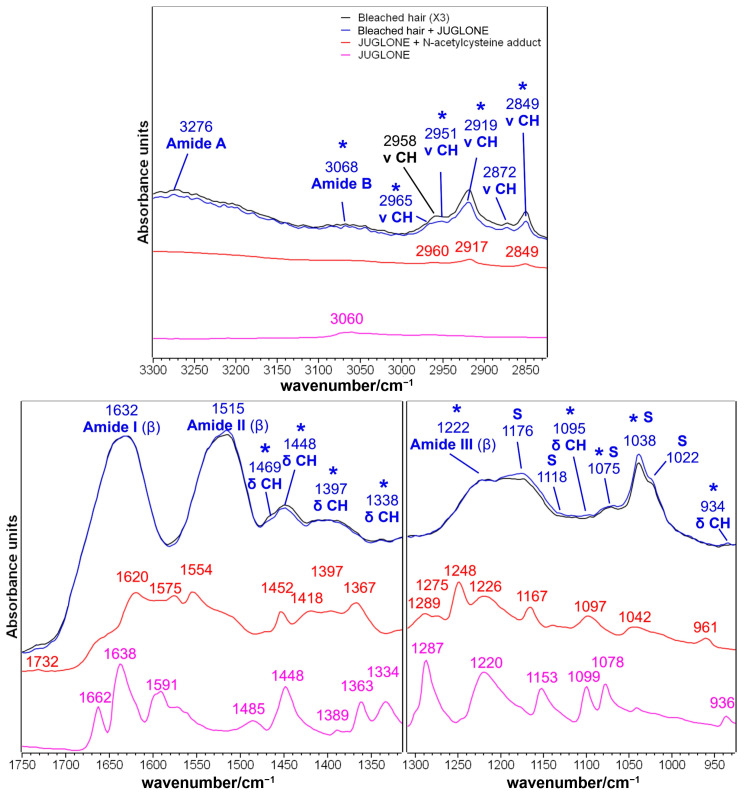
IR spectra of brown hair lock bleached three times (black line) and after the following treatment with a methanolic juglone solution (blue line) in the 3300–2800 and 1750–925 cm^−1^ spectral ranges. Spectra are normalized to the Amide I band. The IR spectra of pure juglone (magenta line) and the thia-Michael addition product between juglone and N-acetyl-L-cysteine (red line) are reported for comparison (see Figure 2 for more details). Bands marked with an asterisk are related to juglone incorporation; S: bands attributed to sulfur oxidation products.

The effects of the subsequent treatment with juglone (Figure 6) can be discussed in comparison with the other repairing agents (such as those based on shikimic acid and maleate), which have in common the same restoring mechanism based on the formation of Michael adducts [10].

In particular, a shikimic acid-based product was found to be incorporated into the cuticle [10], since the IR spectrum allowed the detection of several IR bands attributable to shikimic acid (1114, 1074, and 1040 cm^−1^). Additionally, the treatment rearranged keratin fibers, as demonstrated by the weakening and shifting of the Amide II and III bands, which affected the salt bridges between the SO_3_^-^ groups and ionized basic function (i.e., NH_3_^+^ groups) [10]. Those effects were not observed after the treatment with juglone (Figure 6), which appeared to be more similar to maleate-derived reconstructive agents, which showed only limited effects on the IR spectrum of hair cuticles [10]: only minor modifications were observed for all the Amide bands after the treatment with juglone on hair bleached three times (Figure 6).

The limited incorporation of juglone on this sample can be inferred by the slightly increased absorbance of the bands at 1338, 1095, 1075, 1038, and 934 cm^−1^ and attributable to the quinone or its Michael adduct. Also, sulfur oxidation products showed a moderate increase in the 1176–1022 cm^−1^ spectral region, which was negligible compared with what was observed in hair samples treated with the reducing agent, due to the effects of the previous bleaching treatments. Accordingly, the diagnostic bands attributed to the lipid layer of hair in the 2965–2849 cm^−1^ spectral region (CH stretching vibrations) and 1469 cm^−1^ (CH bending), showed a minor absorbance decrease when compared with the hair samples reduced three times and treated with juglone (Figure 5).

#### 2.1.5. Brown Hair Bleached Three Times after a Reducing Step with NaHSO_3_ and Treatment with Juglone

**Figure 7:**
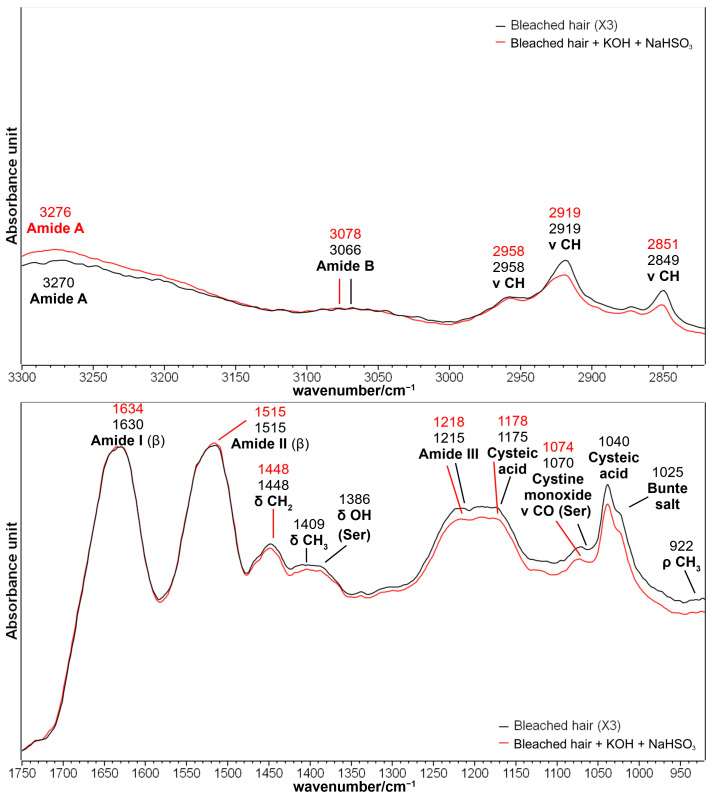
IR spectra of brown hair lock bleached three times (black line) and then treated with KOH and NaHSO_3_ (red line) to simulate hair straightening after bleaching in the 3300–2800 and 1750–900 cm^−1^ spectral ranges. Spectra are normalized to the Amide I band. Abbreviations: β: β-sheet structure; Ser: Serine.

As mentioned in the Section 3, a bleached hair lock was further treated with a solution of KOH at pH = 9 for 5 min and then with a solution of NaHSO_3_ to mimic a straightening treatment following bleaching. Figure 7 shows the effects of these additional treatments.

Generally speaking, only minor variations were detected, mainly in the 1250–900 cm^−1^ spectral region, thus confirming a certain efficiency of the reducing agent towards sulfur oxidation products. Figure S4, Supplementary Materials, shows that the most visible variation in the difference spectrum induced by the reducing treatment was a negative band with a minimum of 1032 cm^−1^, i.e., in the cysteic acid and Bunte salt spectral region. This observation was further confirmed by the statistically significant reduction in the absorbance ratios involving cysteic acid (I_1175_/I_Amide I_ and I_1040_/I_Amide I_), as shown in Table 2.

The other spectroscopic data in the same Table indicate that the treatment induced only minor structural variations, as demonstrated by the I_Amide I_/I_Amide II_ intensity ratio. The difference spectrum in Figure S4, Supplementary Materials, confirmed that the treatment had almost no effect on the Amide I region. At the same time, in the Amide II, a positive band at 1554 cm^−1^ may indicate an increase in cysteine residues coming from the reduction of the disulfide bridges, an effect already observed for the application of sodium hydrogen sulfite on wool fibers [5,54].

**Figure 8:**
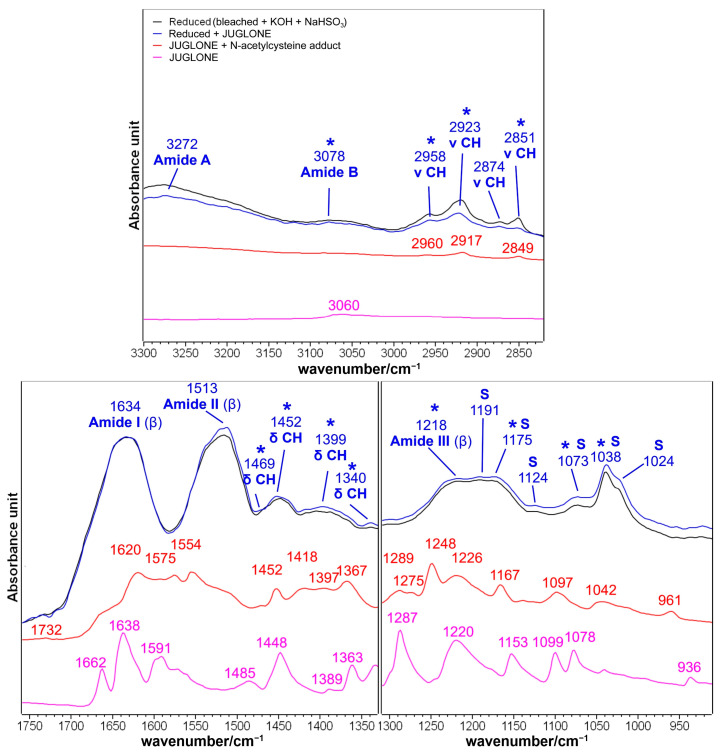
IR spectra of brown hair bleached three times and then reduced with NaHSO_3_ (black line), and after the following treatment with a methanolic juglone solution (blue line) in the 3300–2800 and 1760–910 cm^−1^ spectral ranges. Spectra are normalized to the Amide I band. The IR spectra of pure juglone (purple line) and the thia-Michael addition product between juglone and N-acetyl-L-cysteine (red line) are reported for comparison (see Figure 2 for more details). Bands marked with an asterisk are related to juglone incorporation; S: bands attributed to sulfur oxidation products.

The effects of the treatment with juglone on the hair sample further reduced with sodium hydrogen sulfite are shown in Figure 8.

These effects can be compared with those observed in the three times bleached sample (Figure 6) since it was possible to detect the juglone diagnostic bands at 3078, 1734, 1452, 1399, 1340, 1218, 1175, 1073, and 1038 cm^−1^. The similarities with the bleached samples concerned the following: the increase in sulfur oxidation products (bands at 1191, 1175, 1124, 1073, 1038, and 1024 cm^−1^), the weakening of the bands attributed to the lipid fraction of hair (in the 2950–2800 cm^−1^ CH stretching region and at about 1470 cm^−1^), and minor effects on the structure-sensitive bands such as Amide I, Amide III, and Amide A. More in detail, Figure 8 confirms that after the treatment with NaHSO_3_, the effects of juglone increased and were more evident compared with those observed in the sample bleached three times without the following reducing treatment (Figure 6), mainly in the increased absorbance of the Amide II bands and in the increase in sulfur oxidation products, thus indicating that the increased content of free cysteine residues observed in Figure S4 (Supplementary Materials) may favor the interaction between juglone and hair keratin.

### 2.2. SEM Analyses

The SEM images of the hair fiber subjected to different treatments are herein reported in parallel with the results from the above-reported and discussed IR study.

#### 2.2.1. Brown Hair Treated with KOH, Methyl Thioglycolate, and Juglone

**Figure 9:**
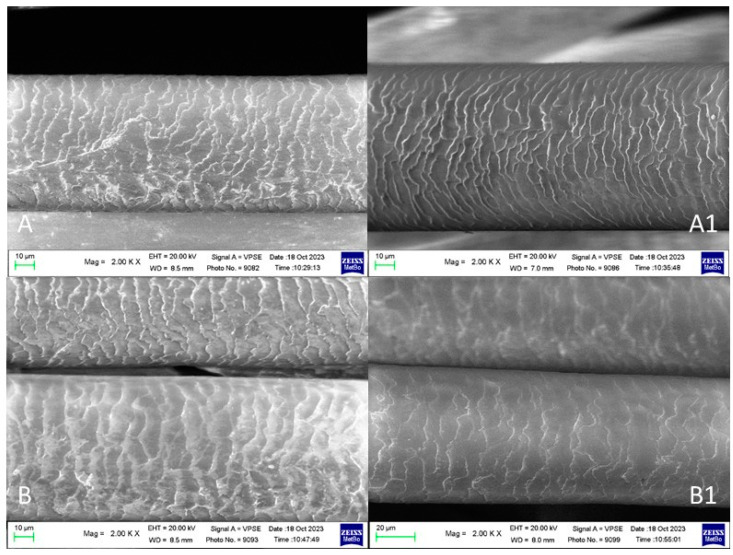
SEM images of virgin brown hair treated with KOH and MT solution for two times (**A**), three times (**B**), and then further treated with a methanolic juglone solution (**A1**,**B1**).

Figure 9 reports the SEM images of virgin brown hair fibers immersed in KOH aqueous solution for 5 min and then in an aqueous solution of MT for two (case A) and three times (case B). The locks were then treated with a methanolic solution of juglone (cases A1 and B1, referred to as control samples A and B, respectively).

As can be seen, after treatment with juglone solution, the hair surface appears more regular, and the scales are more embricated compared with those of the starting samples (compare A with A1 and B with B1, respectively).

The differences between the two consecutive reducing treatments (compare case A with case B) are negligible.

#### 2.2.2. Brown Hair Bleached Three Times and Treated with Juglone

**Figure 10:**
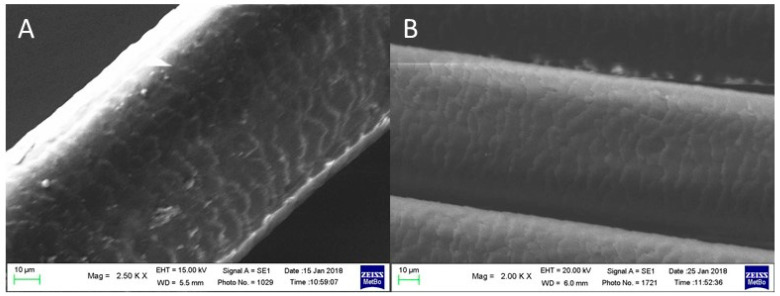
SEM images of: (**A**) hair bleached three times; (**B**) sample further treated with a methanolic solution of juglone.

Figure 10 shows the SEM images of the hair subjected to three bleaching treatments (A) [53] and after the following treatment with a methanolic solution of juglone (B).

After three bleaching treatments (Figure 10A), the hair shows a damaged cuticle with opened scales. The cuticular deterioration significantly decreased after treatment with a methanolic solution of juglone.

#### 2.2.3. Brown Hair Bleached Three Times Treated with KOH, Reducing NaHSO_3_ and Juglone

**Figure 11:**
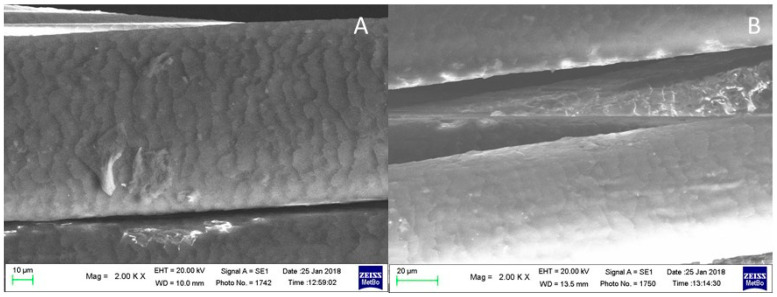
SEM images of: (**A**) hair subjected to KOH and reductive treatment with NaHSO_3_ and (**B**) after the following treatment with a methanolic solution of juglone.

Figure 11 shows the SEM images of the hair bleached three times, immersed in an aqueous KOH solution (pH 9) as a swelling agent, then in an aqueous NaHSO_3_ solution (A), and after the following treatment with the methanolic solution of juglone (B). The hair’s surface appears more regular and smoother after the latter treatment.

## 3. Materials and Methods

### 3.1. Materials

In order to evaluate the modifications that take place on the fiber, following both a bleaching and/or straightening treatment and a reconstructive treatment with juglone, various human hair samples were analyzed. In the first part of the study, juglone was applied to brown hair treated with aqueous KOH at pH = 9 for 5 min since alkaline conditions favor the opening of cuticle scales, facilitating the treatment’s penetration (control sample). A reducing agent, i.e., methyl thioglycolate (in 5% aqueous solution, pH ~9.1), was then applied to the control sample for 5 min up to three times, mimicking the routine operation in hair-straightening treatments. A solution of juglone 5% mol/mol in methanol was prepared by dissolving 0.0865 g of the quinone in 10 mL of methanol and then applied to the treated hair samples at 25 °C for 45 min. Our experiment did not follow the typical hair salon’s practice of applying a neutralizing agent (usually hydrogen peroxide) to restore the disulfide bonds and fix the straight hair conformation. This omission allowed us to maximize the content of free thiol groups to verify if juglone could be used as a restoring agent.

The second part of this study focused on bleaching treatments; therefore, it was decided to start from a previously discoloured and already extensively deteriorated control sample in such a way as to make the subsequent reconstructive treatment more evident at the level of reformed chemical bonds, which can be determined using IR spectroscopy. As a starting material, using a hair sample that has undergone three consecutive bleaching treatments is in line with the approach used in hairdresser saloons [10]. The starting sample was a 10 g lock of hair previously bleached three consecutive times for 45 min at 35 °C with a mixture 1:1 of two commercial products: Lunex Ultra Cream^®^ (based on persulfates) and UniColor Oxi^®^ (oxidizing solution based on hydrogen peroxide) following a consolidated protocol [10,53]. The composition of the two commercial products is described in detail elsewhere [10]. A smaller lock obtained from the control one was treated with the aqueous KOH solution at pH = 9 for 5 min and then immersed in an aqueous solution containing NaHSO_3_ as a reducing agent at pH = 5 for 5 min to mimic the conditions of hair straightening following a decolouration process (breaking of disulfide bridges). After these treatments, the hair lock was immersed in the 5% mol/mol methanolic solution of juglone at 25 °C for 45 min.

### 3.2. IR and SEM Analyses

IR spectra were recorded on five different points for each sample (a lock of hair of about 12 cm length and 0.3 cm diameter) on a Bruker Alpha Fourier Transform FTIR spectrometer equipped with an Attenuated Total Reflectance (ATR) diamond module (penetration depth 2 μm) and a Deuterated Lanthanum α-Alanine-doped TriGlycine Sulfate (DLaTGS) detector; the spectral resolution was set to 4 cm^−1^, and each spectrum equalled an average of 64 scans. Due to their intrinsic orientation, the IR spectra were recorded by positioning the fibers along one specific direction.

The relative content of cysteic acid (as sulfonate salt) was evaluated through the I_1040_/I_Amide_ and I_1175_/I_Amide I_ ratios, where I_1040_, I_1175_, and I_Amide_ were the absorbances (measured as peak heights) of the cysteic acid bands at about 1040 and 1175 cm^−1^ [55] (calculated drawing a baseline between 1330 and 955 cm^−1^) and Amide I, used as an internal standard [10] (calculated drawing a baseline between 1720 and 1350 cm^−1^), respectively. These parameters are essential since cysteic acid is considered the main product of the oxidation of disulfide bonds under the investigated conditions [56]; moreover, increased cysteic acid is considered to be a significant cause of hair damage and local changes in mechanical properties through cysteic acid formation have been presumed to affect the hair texture in an undesirable manner [57].

The Bunte salt/cysteic acid ratio was evaluated through the A_1025_/A_1040_ ratio, where A_1025_ and A_1040_ were the areas of the bands at about 1025 cm^−1^, assignable to the Bunte salt (R-S-SO_3_^−^) [10], and 1040 cm^−1^, assignable to the cysteic acid, respectively. Since these bands can be considered two components of a broader band, areas were determined by a curve fitting procedure after subtracting a baseline in the 1070–950 cm^−1^ interval and using the frequencies of the maxima of the fourth-derivative spectra (obtained with 13-point smoothing) as starting positions for the curve-fitting procedure. The IR component profiles were described as pure Gaussian functions.

Statistical analysis on IR data was performed using R statistical software (version 3.5.3; GNU GPL license). The data have a non-Gaussian distribution, so a non-parametric Kruskal–Wallis test was used for the statistical significance (set at *p* < 0.05), and a Dunn–Bonferroni post hoc analysis was performed for any dependent variable for which the Kruskal–Wallis test was significant. The Kruskal–Wallis test does not compare means but is based on ranks and was used to verify if the rank means are different. Nevertheless, we reported the data as average values with their associated standard deviation (SD) for better readability.

The scanning electron microscopic (SEM) analyses of the surface morphology of human hair samples were performed longitudinally with a Zeiss Evo 50-EP (Carl-Zeiss, Oberkochen, Germany). To minimize artefacts, sputtering was avoided, and the samples were observed in variable pressure (VP) mode. All measures were made at an accelerating voltage of 20 kV and 100 Pa of pressure in the chamber. The signal revealed secondary electrons. For each sample, SEM images were recorded on the central region of the fiber belonging to the same lock; at least two analyses on two different and near regions along the same fiber were performed.

## 4. Conclusions

IR spectroscopy revealed that juglone interacted with hair fibers, modifying the cuticle region, the only one accessible to this vibrational technique. The quinone solution’s effects depended on the previous treatments applied to hair fibers.

In the first part of this study, potent reducing agents, such as MT, which simulate hair straightening, increased free cysteine residues’ content and, therefore, the chemical interaction with juglone. The marker bands of this interaction were attributed by comparison with the IR spectrum of pure juglone and its Michael adduct with N-acetyl-L-cysteine.

**Figure 12:**
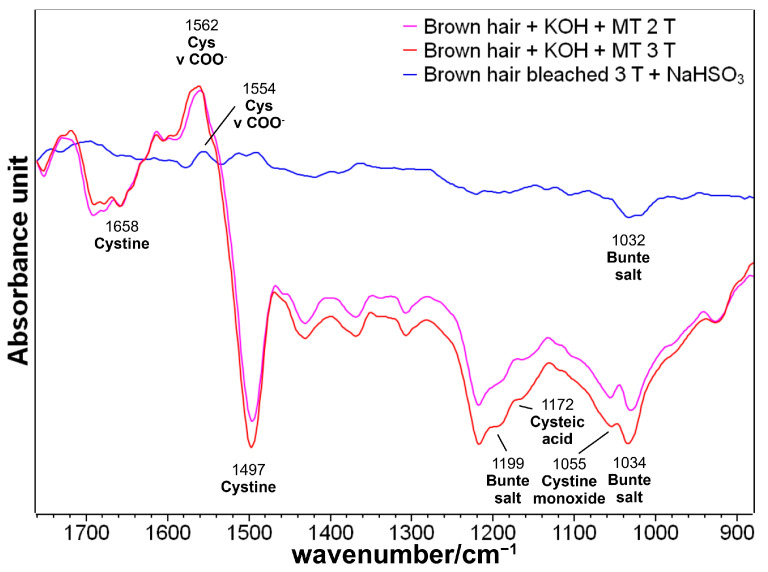
Difference spectra obtained by subtracting from the spectra of the reduced hair samples under study those of the control specimens.

In the second part of this study, hair bleached three times was considered the control sample to check whether the juglone molecule could act as a restoring agent on heavily damaged hair. The quinone interacted to a lower extent compared with hair treated with methyl thioglycolate. The content of the Michael adducts was found to be related to the cysteine content of the samples, which, based on the difference spectra comparatively shown in Figure 12, was found to decrease along the series: brown hair + KOH MT 3 T > brown hair + KOH MT 2 T > bleached hair + KOH + NaHSO_3_. Reducing agents (MT and sodium hydrogen sulfite) favored the reduction of disulfide bonds and increased the content of free cysteine residues, which can react with juglone.

SEM images collected for all the treatments herein reported showed an improvement at the level of the hair surface after treatment with a solution of juglone.

## Figures and Tables

**Table 1:** Values of the I_Amide I_/I_Amide II_, I_1175_/I_Amide I_, I_1040_/I_Amide I_, and A_1025_/A_1040_ ratios (average ± standard deviation) as obtained from the IR spectra of control brown hair (brown hair + KOH) before and after the treatment with methyl thioglycolate (MT) for two and three times (2 T and 3 T). In each column, different letters represent statistically significant differences (*p* < 0.05); NS = not significant.

SAMPLE	I_Amide I_/I_Amide II_	I_1175_/I_Amide I_	I_1040_/I_Amide I_	A_1025_/A_1040_
Brown hair + KOH	1.035 ± 0.005 C	0.126 ± 0.006 A	0.253 ± 0.013 A	0.70 ± 0.06 NS
Brown hair + KOH + MT 2 T	1.082 ± 0.003 B	0.082 ± 0.005 B	0.172 ± 0.011 B	0.61 ± 0.09 NS
Brown hair + KOH + MT 3 T	1.100 ± 0.003 A	0.064 ± 0.004 C	0.147 ± 0.008 C	0.60 ± 0.04 NS

**Table 2:** Values of the I_Amide I_/I_Amide II_, I_1175_/I_Amide I_, I_1040_/I_Amide I_, and A_1025_/A_1040_ ratios (average ± standard deviation) as obtained from the IR spectra of control brown hair bleached three times and after the treatment with KOH and the NaHSO_3_ reductant. In each column, different letters represent statistically significant differences (*p* < 0.05); NS = not significant.

SAMPLE	I_Amide I_/I_Amide II_	I_1175_/I_Amide I_	I_1040_/I_Amide I_	A_1025_/A_1040_
Brown hair bleached 3 T	0.930 ± 0.007 NS	0.317 ± 0.005 A	0.388 ± 0.012 A	0.78 ± 0.10 NS
Bleached 3 T + KOH + NaHSO_3_	0.940 ± 0.009 NS	0.299 ± 0.005 B	0.352 ± 0.011 B	0.74 ± 0.08 NS

## Data Availability

Data are contained within the article or supplementary material.

## Supplementary Materials

The following supporting information can be downloaded at: https://www.mdpi.com/article/10.3390/molecules29020320/s1, Figure S1. IR spectra of brown hair lock before (black line) and after the treatment with aqueous KOH (pH 9) for 5 min (blue line) in the 3500–2800 and 1750–950 cm^−1^ spectral ranges. The red line represents the difference spectrum. Abbreviations: β: β-sheet structure; Ser: Serine; Figure S2. IR spectra of brown hair lock after the treatment with aqueous KOH (pH 9) for 5 min (black line) and the following reduction with methyl thioglycolate (MT) for two and three times (2 T and 3 T, magenta and red lines, respectively) in the 1760–1470 and 1310–950 cm^−1^ spectral ranges. The difference spectrum (red dashed line) between the sample treated three times with MT and the control (KOH-treated brown hair) better shows the differences induced by the reducing treatment. Spectra are normalized to the Amide I band. Abbreviations: β: β-sheet structure; Ser: Serine; Figure S3. Scheme of the Michael addition reaction between the amino end of lysine and juglone [51]; Figure S4. IR spectra of brown hair lock bleached three times (black line) and after an additional KOH + NaHSO_3_ treatment to simulate hair straightening (red line) in the 1750–1360 and 1300–950 cm^−1^ spectral ranges. The difference spectrum (red dashed line) better shows the differences induced by the reducing treatment. Spectra are normalized to the Amide I band. Abbreviations: β: β-sheet structure; Ser: Serine; Table S1. Assignments of the main IR bands of Juglone, N-acetyl-L-Cysteine, and their adducts. Interpretation of vibrations: ν = stretching, δ = bending, ar = aromatic ring; Table S2. Chemical structure of sulfur compounds discussed in the main text.
