# Supplementary material for: Interactions between Damaged Hair Keratin and Juglone as a Possible Restoring Agent: A Vibrational and Scanning Electron Microscopy Study
Source: Molecules. 2024 Jan 9;29(2):320. doi: 10.3390/molecules29020320 (PMC10819223; doi:10.3390/molecules29020320)
Supplement: Supplementary file 1 [file molecules-29-00320-s001.zip › molecules-2749025-supplementary.pdf]

Supplementary Materials

Interactions between damaged hair keratin and juglone as a possible restoring agent: a vibrational and scanning electron microscopy study

Michele Di Foggia [1], Paola Taddei [1,*], Carla Boga [2], Benedetta Nocentini [3] and Gabriele Micheletti [2]

[1] Department of Biomedical and Neuromotor Sciences, Alma Mater Studiorum - Università di Bologna, via Irnerio 48, 40126 Bologna, Italy; paola.taddei@unibo.it, michele.difoggia.2@unibo.it
[2] Department of Industrial Chemistry 'Toso Montanari', Alma Mater Studiorum - Università di Bologna, via Piero Gobetti 85, 40129 Bologna, Italy; carla.boga@unibo.it, gabriele.micheletti3@unibo.it
[3] Kemon S.p.A., Via Enrico Mattei 35, 06016 San Giustino (Perugia), Italy; Benedetta.nocentini@gmail.com
* Correspondence: paola.taddei@unibo.it; Tel.: +39 051 2094280

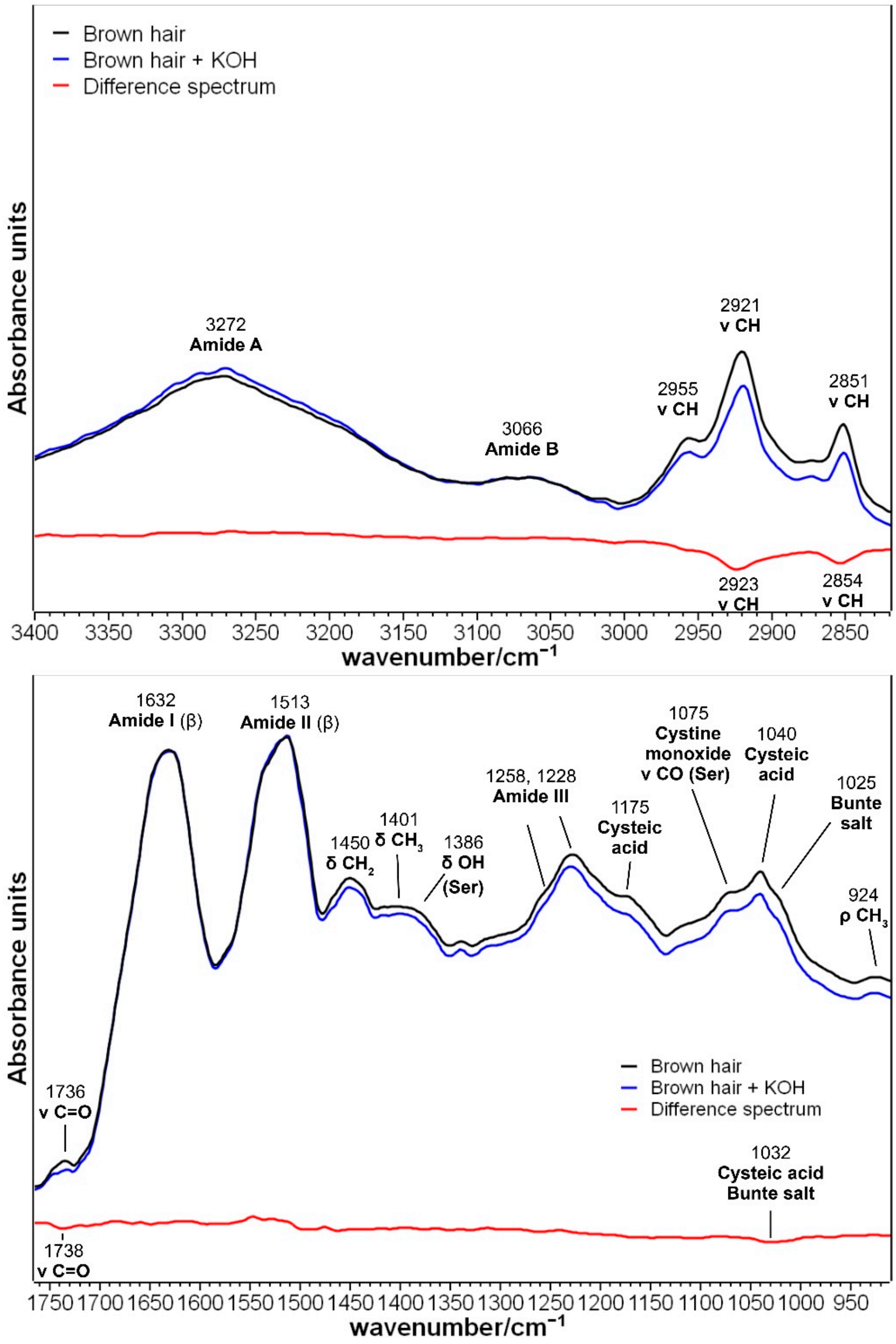

Figure S1. IR spectra of brown hair lock before (black line) and after the treatment with aqueous KOH (pH 9) for 5 minutes (blue line) in the 3500-2800 and 1750-950 cm^{-1} spectral ranges. The red line represents the difference spectrum. Abbreviations: β: β-sheet structure; Ser: Serine.

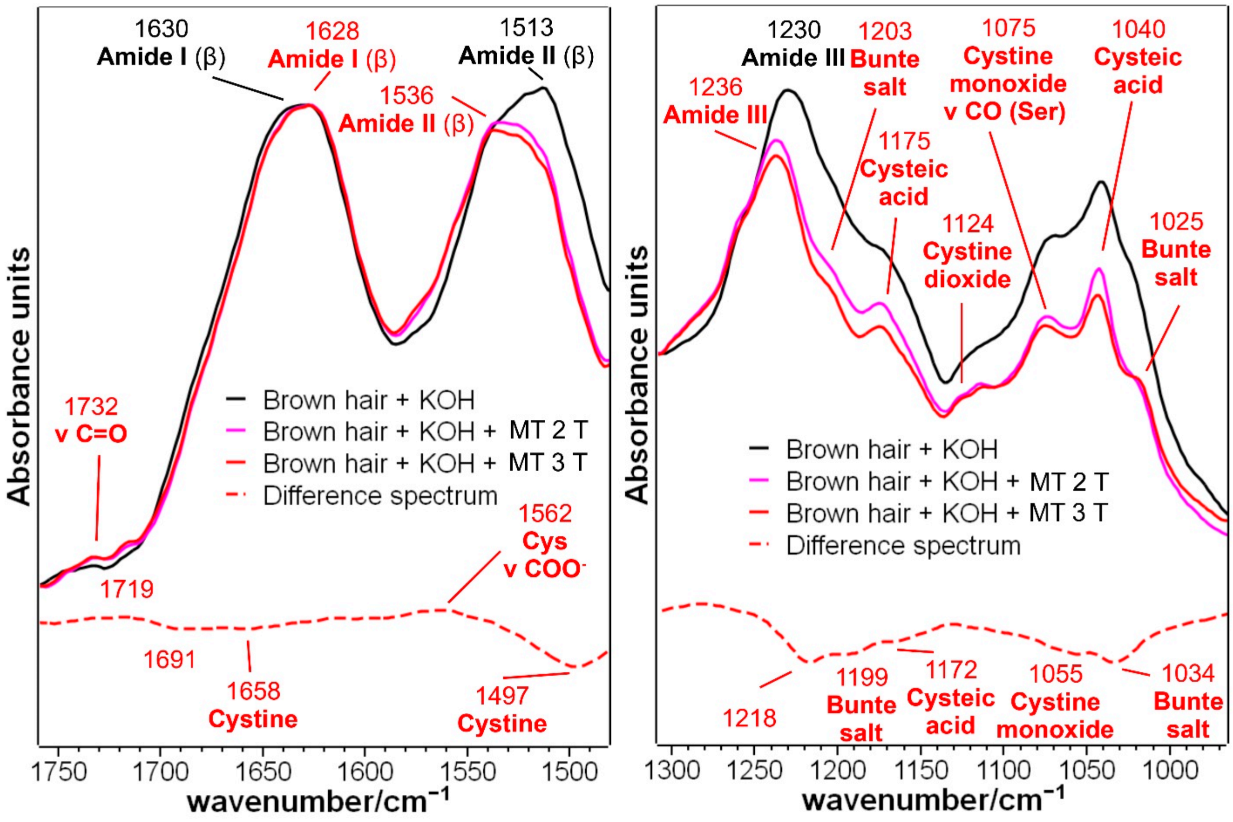

Figure S2. IR spectra of brown hair lock after the treatment with aqueous KOH (pH 9) for 5 minutes (black line) and the following reduction with methyl thioglycolate (MT) for two and three times (2 T and 3 T, magenta and red lines, respectively) in the 1760-1470 and 1310-950 cm^{-1} spectral ranges. The difference spectrum (red dashed line) between the sample treated three times with MT and the control (KOH-treated brown hair) better shows the differences induced by the reducing treatment. Spectra are normalized to the Amide I band. Abbreviations: β: β-sheet structure; Ser: Serine.

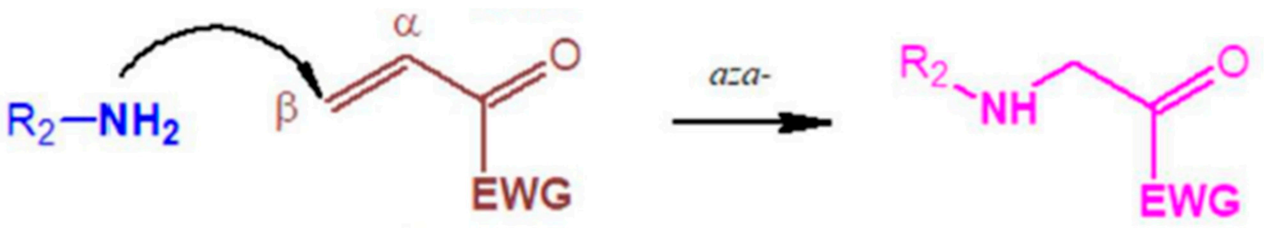

Figure S3. Scheme of the Michael addition reaction between the amino end of lysine and juglone (Chu et al., 2021).

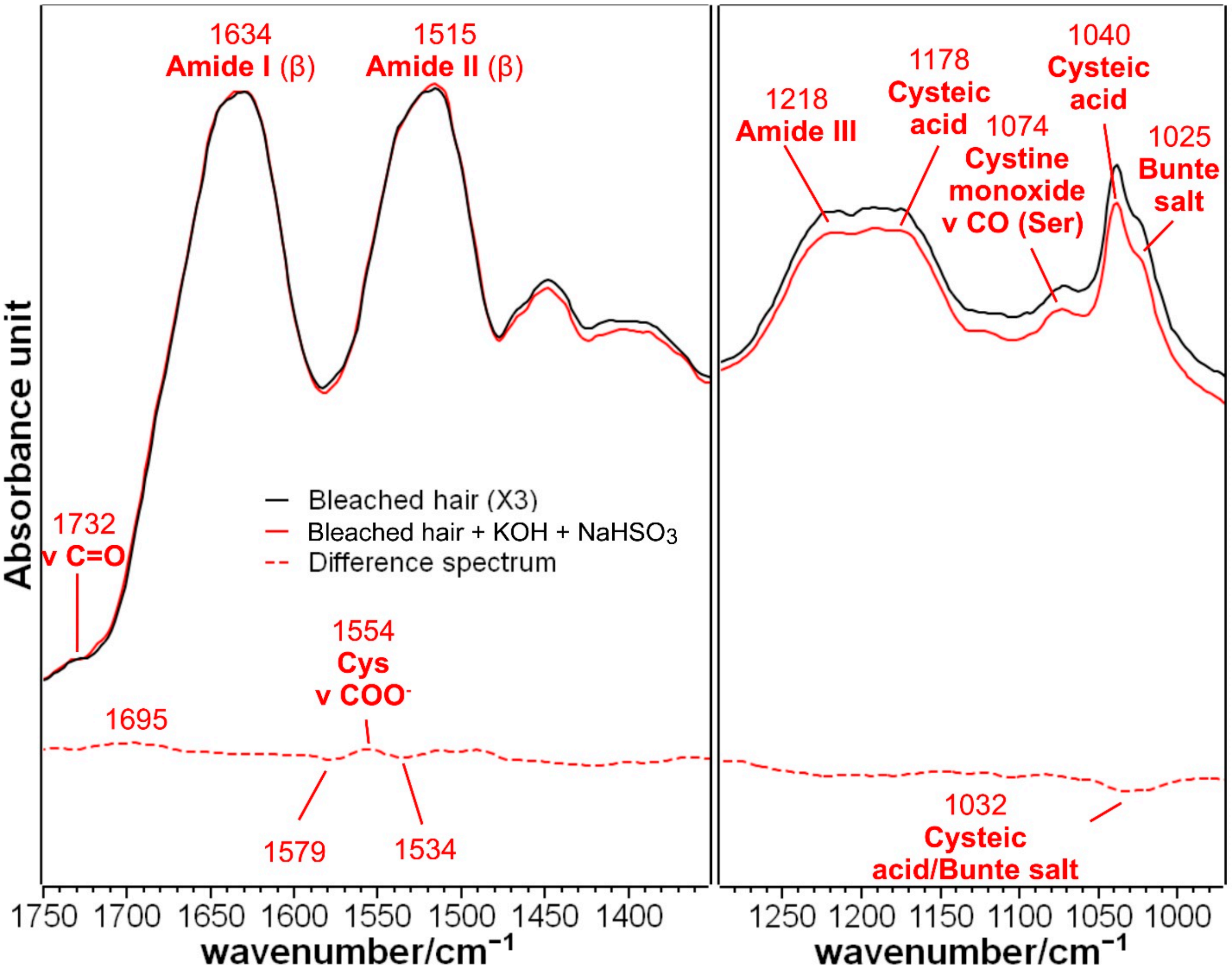

Figure S4. IR spectra of brown hair lock bleached three times (black line) and after an additional KOH + $NaHSO_3$ treatment to simulate hair straightening (red line) in the 1750-1360 and 1300-950 cm^{-1} spectral ranges. The difference spectrum (red dashed line) better shows the differences induced by the reducing treatment. Spectra are normalized to the Amide I band. Abbreviations: β: β-sheet structure; Ser: Serine.

Table S1. Assignments of the main IR bands of Juglone, N-acetyl-L-Cysteine and their adducts. Interpretation of vibrations: ν = stretching, δ = bending, ar = aromatic ring.

Band assignment	Band position/cm^{-1}		
	JUGLONE	N-ACETYL-L-CYSTEINE	ADDUCTS
$\nu_{NH\ free}$ [27,29]		3374	
$\nu_{CH\ ar}$ [19]	3070<br>3060<br>3042		
ν_{CH} [27,29]		2964<br>2898	2960<br>2917<br>2849
$\nu_{SH\ H\text{-}bond}$ [27,29]		2546	
$\nu_{OH\ H\text{-}bond}$ [29]		2430	
$\nu_{C=O\ carboxylic}$ [29]		1907	
$\nu_{C=O\ carboxylic}$ [29]			1732
$\nu_{C=O\ weak\ H\text{-}bond}$ [27,29]		1713	
$\nu_{C=O\ free}$ [19]	1662		
Amide I [29]<br>$\nu_{C=O\ quinone}$ [19]			1648<br>1620
$\nu_{C=O\ H\text{-}bond}$ [19]	1638		
$\nu_{C=C\ ar}$ [25,32]	1591<br>1572		1575
Amide I [29]		1575	
Amide II [29]		1530	1554<br>1522
$\nu_{C=C}$ [18]	1485		1522
$\delta_{C\text{-}H}$ [20,27,31]	1448	1455	1452
$\delta_{C\text{-}H}$ [20,27,31]; ν_{COO^-} [31]		1428<br>1411	1418<br>1396
$\delta_{C\text{-}H}$ [22,27,34]	1363	1371	1367
$\delta_{O\text{-}H}$ [24]	1334		
$\delta_{C\text{-}H}$ [27]		1301<br>1275	
$\nu_{C\text{-}OH}$ [19]	1287		
Amide III [29]			1289
Amide III [27,29]		1252<br>1226	1248
$\delta_{C\text{-}H}$ [31]<br>$\nu_{C\text{-}O}$ [34]			1248
$\delta_{C\text{-}OH}$ [19]	1220		
$\delta_{C\text{-}H}$ [31]			1226
$\nu_{C\text{-}O}$ [27]		1197	
$\delta_{C\text{-}H}$ [31]			1167
$\nu_{C\text{-}C}$ [25]	1153		
$\nu_{N\text{-}C\alpha}$ + $\nu_{C\text{-}O}$ [27]		1126	
$\delta_{C\text{-}H\ ar}$ [32]	1099		
$\nu_{C\text{-}C}$ [31]<br>$\delta_{C\text{-}H\ ar}$ [32]			1097
$\delta_{C\text{-}H\ ar}$ [32]	1078		

δ_{C-H} [31]			1042
δ_{C-H} [27]<br>δ_{C-OH} [31]		1036	
δ_{C-SH} [27]		1008	
δ_{C-H} [27]<br>ν_{C-C} [31]		987	
δ_{C-H} [25,32]			961
ν_{C-C} [27]		940<br>902	
$\delta_{C-H\ ar}$ [26,32]	936<br>857<br>834		855<br>826
δ_{C-SH} [27]		793	
δ_{C-H} [27]<br>δ_{C-SH} [31]		767	
$\delta_{C-H\ ar}$ [32]	744		
ν_{C-S} [34]<br>$\delta_{C-H\ ar}$ [34]			744
$\delta_{=C-H}$ [19]	698		
δ_{C-N} [31]		696	
δ_{C-H} [32]			694
ν_{C-S} [34]		675	669
δ_{C-S} [27,31]		649	653
$\delta_{C-C\ ar}$ [19]	626		
Amide VI [28]		559	
Amide IV [28]		538	538
δ_{C-OO-} [28]		492	
$\delta_{C-C\ ar}$ [19]	461		458
ring torsion [22]	420		430

Table S2. Chemical structure of sulfur compounds discussed in the main text.

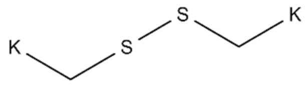	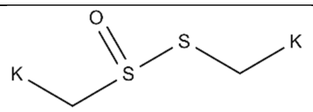	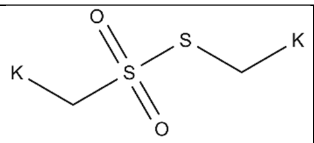	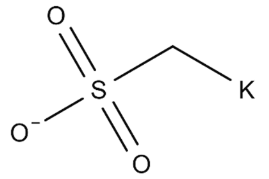
Cystine	Cystine monoxide	Cystine dioxide	Cysteic acid as sulphonate salt

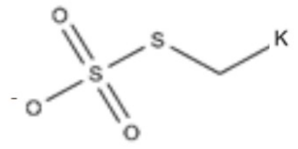
Bunte salt
